# Effects of H_2_SO_4_, GA_3_, and cold stratification on the water content, coat composition, and dormancy release of *Tilia miqueliana* seeds

**DOI:** 10.3389/fpls.2023.1240028

**Published:** 2023-11-09

**Authors:** Yu Wu, Wen Hui Huang, Chen Yin Peng, Yong Bao Shen, Anne M. Visscher, Hugh W. Pritchard, Qiu Gao, Xiao Rui Sun, Ming Zhu Wang, Zhiyun Deng

**Affiliations:** ^1^ College of Forestry, Nanjing Forestry University, Nanjing, Jiangsu, China; ^2^ Co-innovation Center for Sustainable Forestry in Southern China, Southern Tree Inspection Center National Forestry Administration, Nanjing, Jiangsu, China; ^3^ Trait Diversity and Function Department, Royal Botanic Gardens, Kew, Wakehurst, Ardingly, West Sussex, United Kingdom; ^4^ Kunming Institute of Botany, Chinese Academy of Sciences, Kunming, Yunnan, China; ^5^ Ministry of Agriculture and Rural Affairs of the People’s Republic of China, National Animal Husbandry Service, Beijing, China

**Keywords:** H2SO4-GA3 treatment, water gap and channels, mechanical properties, seed coat components, dormancy release

## Abstract

**Introduction:**

*Tilia miqueliana* is an endemic species whose population is declining. The permeability barrier and mechanical constraint of the pericarp (seed coat) are important causes of its seed dormancy. Although there has been considerable research on this subject, questions remain regarding how the permeability barrier and mechanical constraint of the seed coat are eliminated during dormancy release and how water enters the seed. Therefore, protecting the species by improving its germination/dormancy breaking in the laboratory is urgent

**Methods:**

In this study, the changes in the cellular structure, mechanical properties, and components of the *Tilia miqueliana* seed coat after an H_2_SO_4_-gibberellic acid (GA_3_) treatment were analyzed during dormancy release. Various analyses (e.g., magnetic resonance imaging, scanning electron microscopy, and paraffin section detection) revealed the water gap and water channel.

**Results:**

The H_2_SO_4_ treatment eliminated the blockage at the micropyle and hilum of the seeds. Water entered the seeds through the water gap (micropyle) rather than through the hilum or seed coat, after which it dispersed along the radicle, hypocotyl, and cotyledon to the endosperm. During the cold stratification period, the cellular structure was damaged and an increasing number of holes appeared on the inner and outer surfaces of the seed coat. Vickers hardness tests showed that GA_3_ decreased the seed coat hardness. Additionally, the seed coat lignin and total phenol contents continuously decreased during the cold stratification period. Notably, the Liquid chromatography–mass spectrometry (LC–MS) analysis of the seed coat detected polyethylene glycol (osmoregulator), which may have destabilized the water potential balance inside and outside the seed and increased the water content to levels required for germination, ultimately accelerating seed dormancy release.

**Discussion:**

This sophisticated and multi-level study reveals how H_2_SO_4_ and GA_3_ eliminate the permeability barrier and mechanical constraints of the seed coat during dormancy release of *Tilia miqueliana* seeds. This will be beneficial to artificially assist the natural regeneration and population expansion of *Tilia miqueliana*.

## 1 Introduction

Physical dormancy (PY) is caused by a water-impermeable seed or fruit coat (Baskin et al., 2000). The impermeable palisade tissue of the seed coat is often composed of stone cells and has a thick lignified secondary cell wall. The cell wall is filled with hydrophobic substances, including keratin, lignin, quinones, pectin, cork, and wax, which prevent water molecules from entering the seed or fruit cavity (Sahai and Pal, 1995). The seed coat also contains water channels (e.g., micropyle, hilum, and chalaza) that form a special water-gap structure to prevent water from entering (Gama-Arachchige et al., 2011). A water-gap structure, which is an important component of physically dormant seeds, has been identified in 12 of the 16 families that produce physically dormant seeds (Baskin et al., 2000; Baskin et al., 2006; Jayasuriya et al., 2007). Bulges adjacent to the micropyle function as the water gap in other *Convolvulaceae* species with physically dormant seeds. For example, the hilar fissure serves as a water gap in *Cuscuta* species (Jayasuriya et al., 2008). Under natural conditions, PY can be broken by several factors, including high and low temperatures, heat shock, fire, animals, and microorganisms (Bakker, 2001; Briggs and Morris, 2008). Hard-seed populations of *Stylosanthes humilis* and *Stylosanthes hamata* soften when the maximum soil surface temperature exceeds 50°C–55°C (McKeonVan et al., 1982). The dormancy of the hard seeds of 11 *Geraniaceae* and *Malvaceae* species under natural conditions may be broken by drying in summer, by specific temperature regimens, or by the gradual softening of the seed coat, thereby ensuring germination occurs over many seasons (Van Assche and Vandelook, 2006). Although PY has been confirmed in *Albizia julibrissin*, the dormancy of seeds immersed in hot water for 30 s may be broken because of the formation of a circular lid-like opening at the lens, ultimately leading to germination (Yang et al., 2020). The PY of *Townsville stylo* seeds decreases in response to scarification or treatments with acidic solutions. Seeds must overcome PY prior to radicle protrusion (Chaves et al., 2017). In addition to the permeability barrier, the mechanical constraint imposed by the seed coat is also an important cause of seed dormancy. Mechanical dormancy (MD), but not PY, is responsible for the poor germination of teak (*Tectona grandis* Lf) seeds (Slator et al., 2013). The germination rate and dormancy-breaking effects are directly related to the degree of cuticle breakage and disintegration at the surface of *Sorbus amabilis* seeds (Chen et al., 2011). The seed coat is reportedly a limiting factor for the germination of *Sorbus pohuashanensis* seeds (i.e., the naked embryo germination rate is significantly higher than the intact seed germination rate) (Yang et al., 2012).

*Tilia miqueliana* Maxim, which is an endemic species in the family *Tiliaceae*, is distributed in eastern China, but its population is gradually declining because of the difficulty in its natural regeneration (Shi et al., 2008). The dormancy of *T. miqueliana* seeds is due to the PY and MD of the pericarp and seed coat and the physiological dormancy (PD) of the endosperm (Wang, 2007). Notably, *T. miqueliana* fruits/seeds are only partially impermeable. Specifically, water can enter the fruit and seed cavity through the fruit handle hole and micropyle (Wu and Shen, 2021a; Wu and Shen, 2021b). In this study, the mechanism through which H_2_SO_4_ and gibberellic acid (GA_3_) eliminate the permeability barrier and mechanical constraint of the seed coat during the breaking of *T*. *miqueliana* seed dormancy was investigated. The study findings suggest that H_2_SO_4_ is critical for breaking the permeability barrier of *T. miqueliana* seeds. Moreover, GA_3_ is important for enhancing endosperm hydration, weakening the seed coat, and accelerating the metabolism of seed coat components. The imbibition of hard-coat seeds is commonly inhibited, but imbibition is the first and vital step of seed dormancy release. Thus, the results of this study provide new insights into the water gap and channels, hydration, and the mechanical properties and components of hard-coat seeds.

## 2 Materials and methods

### 2.1 Plant material and seed treatments

*Tilia miqueliana* fruits were harvested in November 2020 at the Huang Zangyu National Forest Park (117°03′–117°06′E, 34°–34°06′N), Anhui province, China. Fruits with full seeds underwent an X-ray image analysis. Fruits were placed on No. 3 enlarging paper and analyzed using an HY-35 X-ray machine (Xiang Xi Instrument Factory, Hunan, China), with the following settings: voltage, 80 kV; current, 5 mA; exposure time, 120 s; and focus-film distance, 25 cm. Seeds were obtained by manually hulling fruits and then stored at 4°C.

The following treatment groups were included in this study: H_2_SO_4_, H_2_SO_4_-GA_3_, peeled, and control (untreated). The treatments were completed using three replicates, each comprising 3,000 seeds, with the exception of the peeling treatment, which was performed using replicates consisting of 100 seeds. The control seeds were soaked in distilled water for 12 h. The seeds in the H_2_SO_4_ treatment group were soaked in 98% H_2_SO_4_ for 15 min and then in distilled water for 12 h, whereas the seeds in the H_2_SO_4_-GA_3_ treatment group were soaked in 98% H_2_SO_4_ for 15 min and then in 500 mg L^−1^ GA_3_ for 12 h. For both treatments, the seeds were mixed with wet sand (45% humidity) at a ratio of 1:3 and stratified at 4°C for 75 days. For the seeds in the peeled treatment group, the seed coat was removed by hand.

### 2.2 Germination test

Three replicates of 100 seeds each were included in control, H_2_SO_4_, and H_2_SO_4_-GA_3_ treated groups. The seeds were mixed with wet sand (45% humidity) at a ratio of 1:3 and stored in a cold storage (4°C) without light for germination testing over a period of 75 days. When the radicle length reached 2 mm, the seeds were considered to have germinated. Percent germination was calculated using the formula proposed by Gairola et al. (2011): $G=\frac{N}{A}\;\times 100$ , where *G* = % germination, *N* = number of embryos germinated, and *A* = total number of embryos tested. Final percent germination represents the mean (± standard deviation) of three replicates.

### 2.3 Water content during imbibition and cold stratification

The water content of the control, H_2_SO_4_, and H_2_SO_4_-GA_3_ treatment groups was measured using a gravimetry-based method at selected time points during the imbibition and cold stratification periods. The seeds in the imbibition group were soaked in distilled water, while the seeds in the cold stratification group were placed in wet sand. For each group, three replicates of 100 seeds were placed in a 50-mL beaker containing water for an imbibition at 25°C in a temperature-controlled lightbox. Seeds were weighed at regular intervals until saturated (i.e., constant weight). The increase in the water content (%) was calculated using the following formula as described by Guo et al. (2018): $\;\text{Water content}=\frac{Wt\;-\;Wo}{Wo}\;\times \;100$ , where *Wt* is the weight of the imbibed seeds at time *t* and *Wo* is the initial seed weight. The sampling time points were 0.5, 4, 10, 20, 30, 40, 50, 60, 70, 80, 120, 140, 160, and 180 h for the imbibition period and 0.5, 15, 30, 45, 60, and 75 days for the cold stratification period. After being examined at each time point, the seeds were returned to distilled water or wet sand.

### 2.4 Water distribution during imbibition and cold stratification

The water distribution of the control, H_2_SO_4_, and H_2_SO_4_-GA_3_ treatment groups was tested *via* the ^1^H MRI analysis of the H protons (water protons) in water molecules, which, in seeds, may be classified as free or bound. Because bound water cannot directly generate a signal, the ^1^H MRI signal represents free water. The three treatment groups were analyzed during the imbibition and cold stratification periods. The seeds that underwent the imbibition treatment were soaked in distilled water, whereas the seeds that underwent the cold stratification treatment were placed in wet sand. Eight seeds were included in each treatment group. After the examination at each sampling time point, the seeds were returned to the distilled water or wet sand. The sampling time points were 0.5, 4, 10, 20, 30, 40, 50, 60, 70, 80, 120, 140, 160, and 180 h for the imbibition treatment and 0.5, 15, 30, 45, 60, and 70 days for the cold stratification treatment.

A high-field nuclear MRI apparatus (7.0 T, PharmaScan; Biospin GmbH, Bruker, Rheinstetten, Germany) was used to track the channel of free water entering the seeds and the water distribution within the seeds. Using a 45-mL centrifuge tube, drupes and their respective seeds were fixed on 25 × 75 mm microscope slides, after which they were placed in the volume coil and analyzed at a resonance frequency of 300.337 MHz at 22°C ± 1°C. The proton intensity MR images were recorded using turbo-rapid acquisition with a relaxation-enhancement proton density-weighted sequence (repetition time/echo time, 1,033/10 ms; slices, 27; field of view, 2.8 × 2.8 cm; number of averages, 18; matrix, 256 × 256; slice thickness/gap, 0.3/0 mm; and flip angle, 180°). Anatomical images were captured with a pixel resolution of 109 µm and a scan time of 15 min. Time-lapse images were captured continuously at 23-s intervals. Seven images were captured per sample and oriented along the longitudinal axis. All imaging was conducted at 25°C. The spatial distribution of water in the seeds was visualized using false colors along a relative scale from zero (black) to maximum signal strength (white). False color technology was used to transform shades of gray in black-and-white images into different colors according to linear or non-linear mapping functions. Gray bytes in the original image f (x,y) were in three groups corresponding to red (R), blue (G), and yellow (B) codes; each group was transformed into three primary color components *via* D/A conversion. ImageJ was used to acquire the MR image slices.

### 2.5 Cellular structure of the seed coat during cold stratification

#### 2.5.1 Scanning electron microscopy

A scanning electron microscopy (SEM) system (FEI, Hillsboro, OR, USA) was used to analyze the cell morphology of the micropyle, hilum, and inner and outer surfaces, and the thickness of the seed coat during the cold stratification period. Seeds from the control, H_2_SO_4_, and H_2_SO_4_-GA_3_ treatment groups were sampled during the cold stratification period. The analyzed samples included the micropyle, hilum, and seed coat from eight replicates of 500 seeds. The sampling time points were 0.5, 10, 20, 30, 35, 40, 45, 50, 55, and 60 days during the cold stratification period. Samples were obtained from fruits without damaging the cells, cut into 2-mm^2^ pieces, and mounted on a sample table using double-sided tape. They were then coated in gold using a gold sputter coating tool (E-1010; Hitachi, Tokyo, Japan) and examined using the high vacuum mode of the SEM system. For each sample, seven images were captured at 15 kV. ImageJ was used to analyze and process the SEM images.

#### 2.5.2 Paraffin section detection

The seed coat cell morphologies were examined *via* paraffin section detection (PSD). Seeds from the control, H_2_SO_4_, and H_2_SO_4_-GA_3_ treatment groups were sampled during the imbibition and cold stratification periods. Eight randomly selected seeds from each treatment group were analyzed on day 0.5. Intact seeds were cut longitudinally using a single-sided blade and fixed in FAA (5% formalin and 5% acetic acid in 90% ethanol) fixative solution (Servicebio, Wuhan, China) for 24 h (Joel et al., 2012). The seeds were dehydrated using an ethanol series and then embedded in wax blocks. The blocks were cut into 3-μm thick segments using the glass knife of the RM2016 ultramicrotome (Laika, Shanghai, China) and then stained with safranin and fast green (Ma et al., 1993). The sections were examined using a light microscope (Nikon, Tokyo, Japan). Seven images were captured for each seed. The PSD images were analyzed and processed using the Case-Viewer digital microscope application (https://www.3dhistech.com/caseviewer).

#### 2.5.3 Atomic force microscopy

An atomic force microscopy (AFM) system (Dimension Edge, Bruker, Germany) was used to observe the three-dimensional cytoarchitecture of the outer surface of the seed coat at specific time points during the stratification period (i.e., 0, 50, and 70 days for the H_2_SO_4_ treatment group and 0, 30, and 45 days for the H_2_SO_4_-GA_3_ treatment group). Eight seed coat samples were collected for both treatment groups. The tissue from the outer surface of the seed coat was cut into 1-mm^3^ pieces and fixed immediately in 2.5% (v/v) glutaraldehyde aqueous fixative solution (Servicebio) for 2 h. The samples were treated with 1% (w/v) OsO_4_ for 5 h, dehydrated using an ethanol series, and embedded in resin blocks. The resin blocks were cut using the UC7 ultramicrotome (Leica, Wetzlar, Germany) equipped with a diamond slicing knife (Ultra 45°; Daitome, Bienne, Switzerland). The resin slices were fixed onto slides using a double-sided tape. Atomic-scale topographic images of the sample surface were acquired using a controlled piercing probe at a scan rate of 1.0 Hz and a scan range of 5 µm.

### 2.6 Mechanical properties of the seed coat during the cold stratification period

#### 2.6.1 Vickers hardness

The hardness of the seed coat was measured using a Vickers hardness machine (Falcon 507, Innovatest, Maastricht, The Netherlands). Eight seeds from the H_2_SO_4_ treatment group (0, 50, and 70 days) and the H_2_SO_4_-GA_3_ treatment group (0, 30, and 45 days) were analyzed. After the outer surface of the seed coat was polished, the seed was fixed using a clamp. A rhombus-shaped indentation was pressed into the outer surface of the seed coat using a diamond square pyramid with a vertex angle of 136°. A loading force of 10 gf was maintained for 10 s. The pressure was calculated according to the pressure per unit surface area of the indentation. The length of the two diagonal lines of the indentation was measured using the microscope on the instrument. The hardness value was displayed by the software.

#### 2.6.2 Lignin, cellulose, and hemicellulose contents

A total of 50 seeds were selected from the H_2_SO_4_ treatment group (0, 50, and 70 days) and the H_2_SO_4_-GA_3_ treatment group (0, 30, and 45 days). The cellulose content was determined using an anthrone colorimetric method (DuBois et al., 1956), whereas the lignin content was measured according to an acetyl bromide spectrophotometric method (Fukushima and Hatfield, 2001), and the hemicellulose content was determined by conducting a DNS assay (Deshavath et al., 2020).

### 2.7 Seed coat components during the cold stratification period

We identified the naturally occurring substances in the samples from the H_2_SO_4_ treatment group (0, 50, and 70 days) and the H_2_SO_4_-GA_3_ treatment group (0, 30, and 45 days) by LC-MS using the UltiMate 3000 RS chromatography system (Thermo Fisher Scientific) and the TSQ Quantum mass spectrometer (Thermo Fisher Scientific) (Zhao et al., 2019). For both treatment groups, seven replicates were analyzed for each time point. The seed coat was extracted from 50 intact seeds to account for differences among individual seeds. The metabonomics LC-MS data were deposited in an online database (www.ebi.ac.uk/metabolights/MTBLS7830; unique identifier: MTBLS7830).

Samples were ground to a fine powder, and then, 200 mg of ground material was added to 1 mL methanol:water (8:2) solution, thoroughly mixed by vortexing, and centrifuged at 20,000×*g* for 10 min at 4°C. The supernatant was filtered through a 0.22-µm membrane, and the filtrate was analyzed (Mathon et al., 2013). The LC-MS analytical conditions are outlined in **Table 1**. The high-resolution liquid chromatography data were split using the Compound Discoverer 2.1.0.401 software (Thermo Fisher) and then retrieved and compared with the mzCloud, mzVault, and ChemSpider databases on the basis of the retention value.

**Table 1:** Gas chromatography-mass spectrometry (LC-MS) conditions for analysis of the seed coat in H_2_SO_4_ and H_2_SO_4_-GA_3_ treated group.

Mass Spectrometry Conditions	
Parameter	Conditions
Ion source	Electrospray ionization source (ESI)
Scanning method	Positive and negative ion switching scanning
Detection method	Full mass/dd-MS_2_ detection
Resolution power	70,000 (full mass) and 17,500 (dd-MS_2_) resolution
Scan range	100.0–1,500.0 m/z
Spray voltage	3.8 kV (positive)
Capillary temperature	300 °C
Collision gas	High purity argon (purity ≥99.999%)
Sheath gas	Nitrogen (purity ≥99.999%), 40 Arb
Aux gas and heater temperature	Nitrogen (purity ≥99.999%) at 350°C
Data collection time	27.0 min
Chromatography conditions	
Parameter	Conditions
Column	RP-C18 150 × 2.1 mm, 1.8 um
Flowrate	0.300 mL/min
Aqueous phase	0.1% formic acid aqueous solution
Organic phase	0.1% acetonitrile formate
Needle washing solution	Methanol
Column temperature	35°C
Automatic injector temperature	10°C
Injection needle length	2.00 mm
Autosampler cleaning settings	Both
Automatic injector needle washing volume	200.00 μL
Soaking time for the needle cleaning process	3.00 ms
Autosampler injection volume	10.00 μL

### 2.8 Statistical analysis

**Figure 1:**
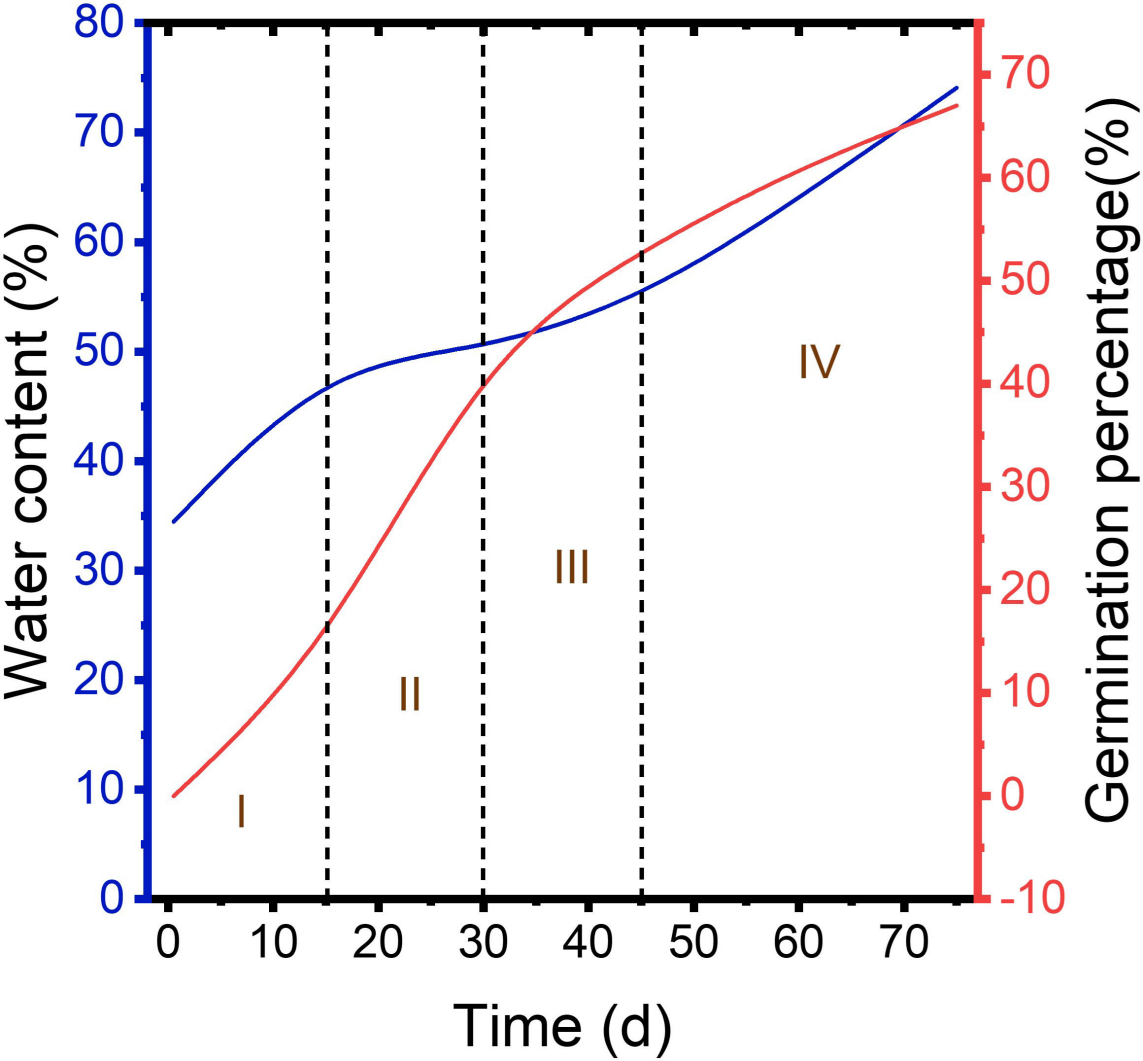
Germination percentage of control, H_2_SO_4_, and H_2_SO_4_-GA_3_ groups during cold stratification process.

**Figure 2:**
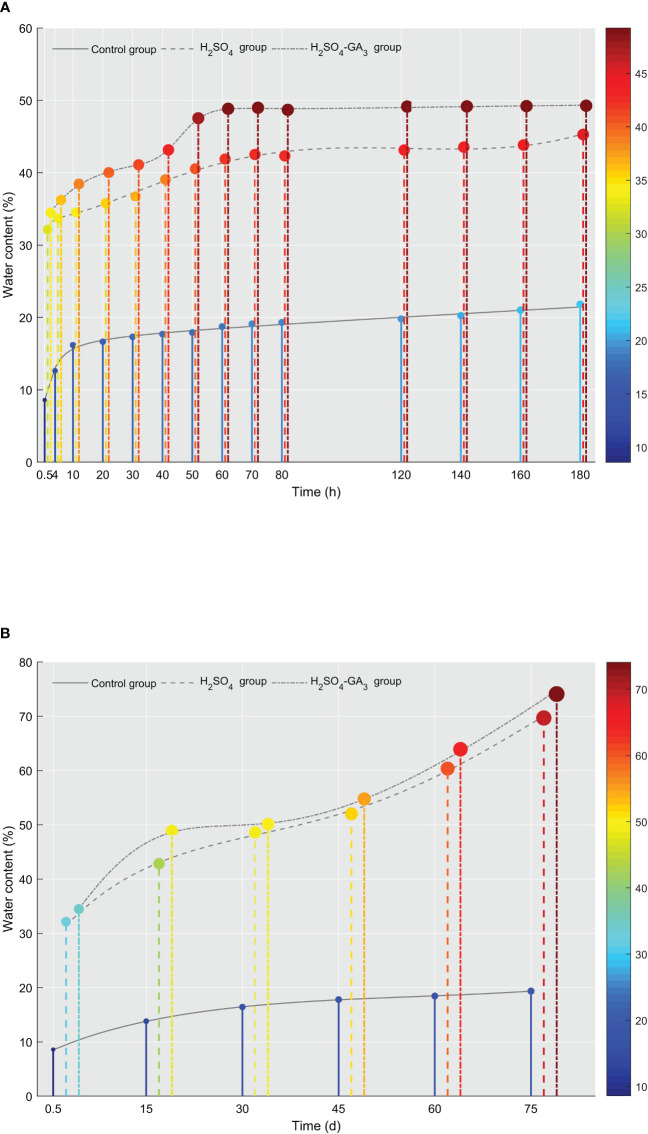
Changes in the water content in the control, H_2_SO_4_, and H_2_SO_4_-GA_3_ treatment groups during the imbibition **(A)** and cold stratification **(B)** periods. The x-axis presents the imbibition and stratification times (h/days), and the y-axis presents the water content (%). The color bar (right) and the corresponding scale values represent the water content (red, high; blue, low).

**Figure 3:**
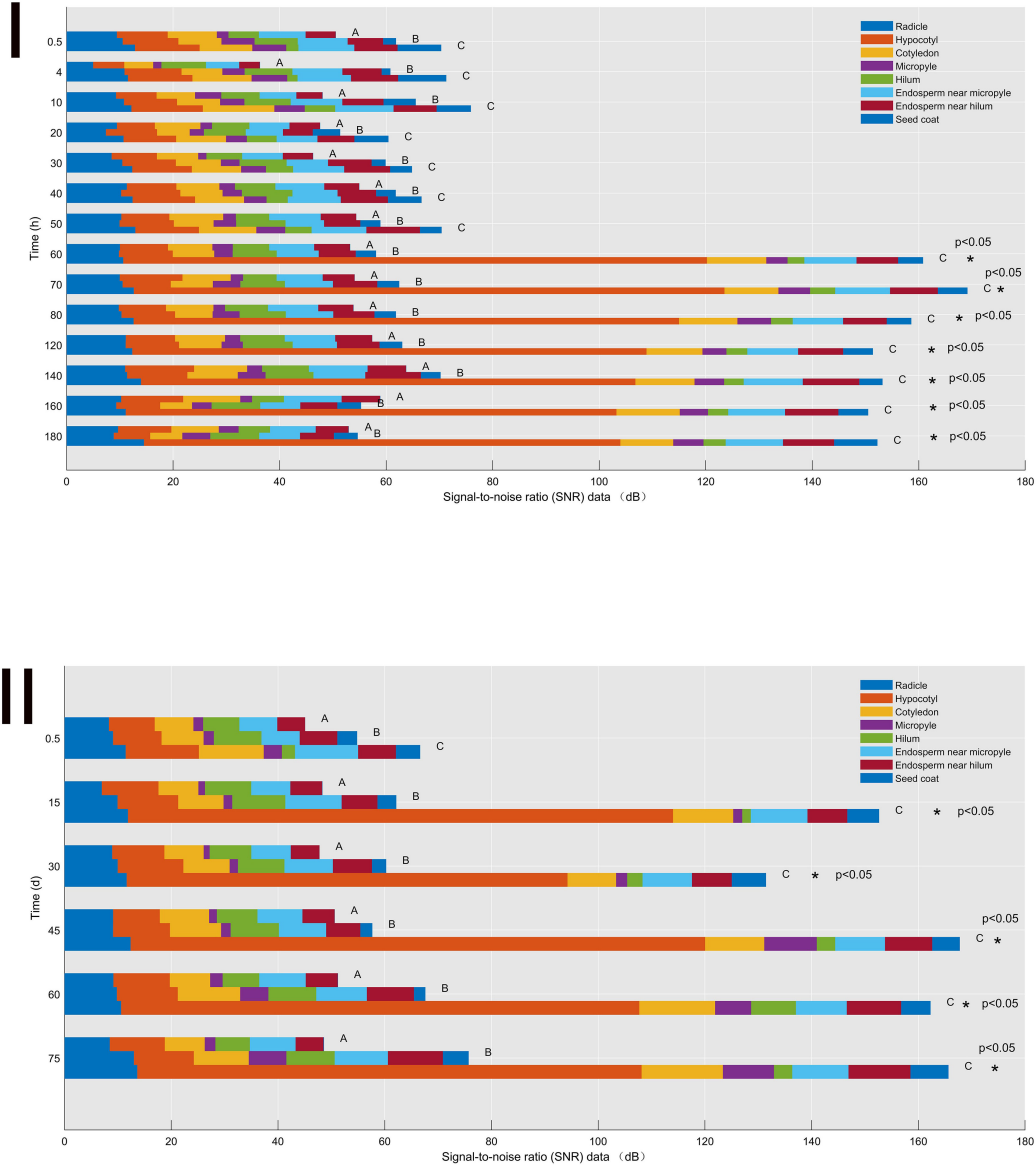
Signal-to-noise ratio (SNR) data of the control (A), H_2_SO_4_ (B), and H_2_SO_4_-GA_3_ (C) treatment groups during the imbibition **(I)** and cold stratification **(II)** periods. Different colors represent SNR data for different sites (radicle, hypocotyl, cotyledon, micropyle, hilum, endosperm near micropyle, endosperm near hilum, and seed coat). The x-axis presents the signal-to-noise ratio (SNR) data, and the y-axis presents the imbibition and cold stratification times (h/days). *Significant differences p< 0.05.

**Figure 4:**
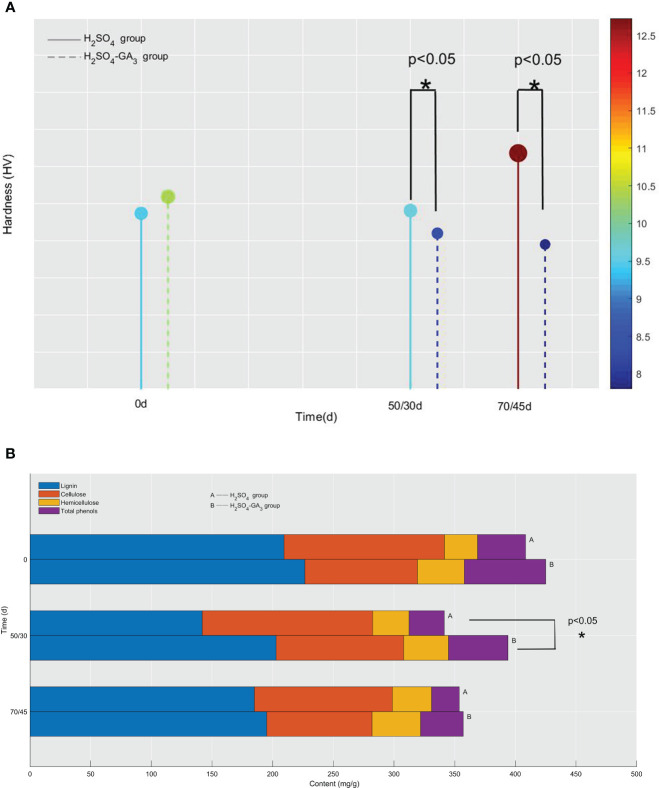
Hardness **(A)** and lignin, cellulose, hemicellulose, and total phenol contents **(B)** of the seed coat in the H_2_SO_4_ group (0, 50, and 70 days) and in the H_2_SO_4_-GA_3_ group (0, 30, and 45 days) during the cold stratification period. In panel **(A)**, the x-axis presents the stratification time (days), and the y-axis presents the hardness (HV). The color bar (right) and the corresponding scale values represent the hardness (red, relatively hard; blue, relatively soft). In panel **(B)**, the x-axis presents the lignin, cellulose, hemicellulose, and total phenol contents (mg/g), and the y-axis presents the stratification time (days).

**Figure 5:**
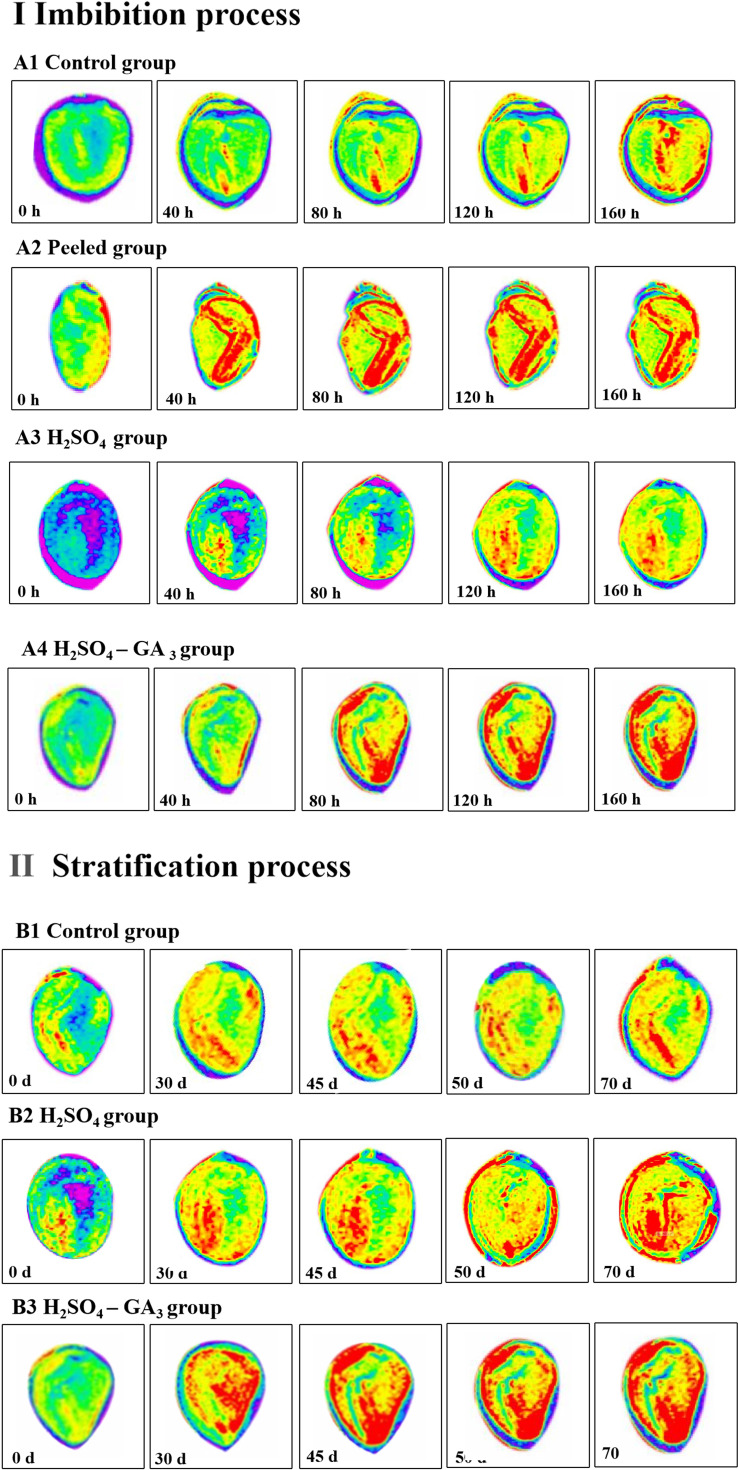
Distribution of water in the seeds of the control (A1, B1), peeled (A2), H_2_SO_4_ (A3, B2), and H_2_SO_4_-GA_3_ (A4, B3) treatment groups during the imbibition and cold stratification periods. Red, blue, and yellow represent the hydrated, impermeable, and hydrophobic regions of the seed tissue, respectively.

**Figure 6:**
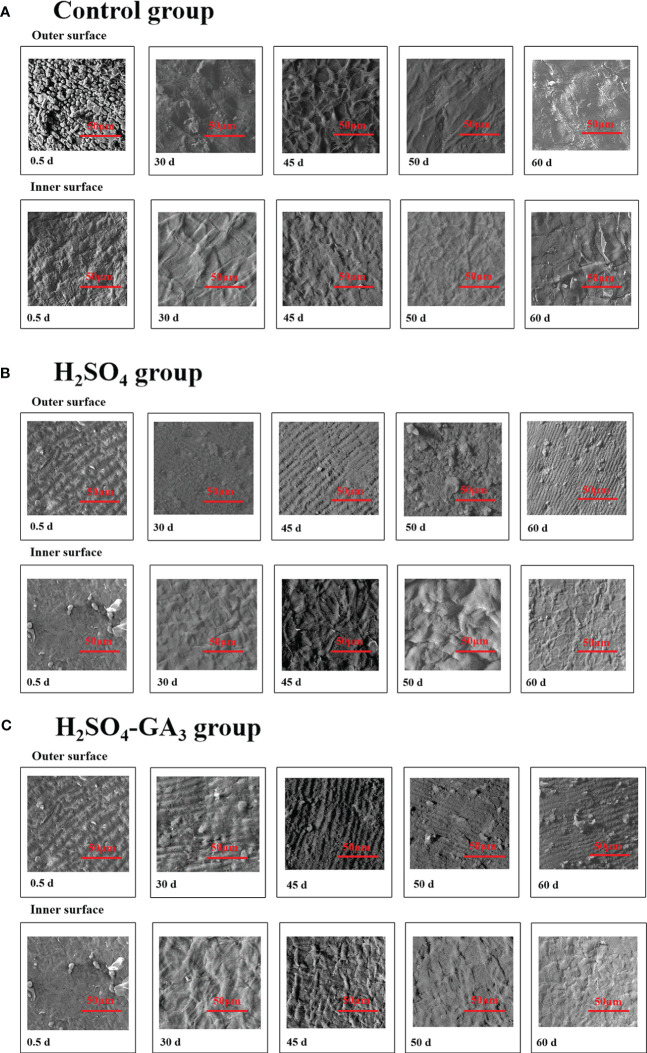
Two-dimensional cellular structure of the outer surface of the inner layer of the seed coat in the control (A1, A2), H2SO4 (B1, B2), and H2SO4-GA3 (C1, C2) treatment groups during the cold stratification period.

**Figure 7:**
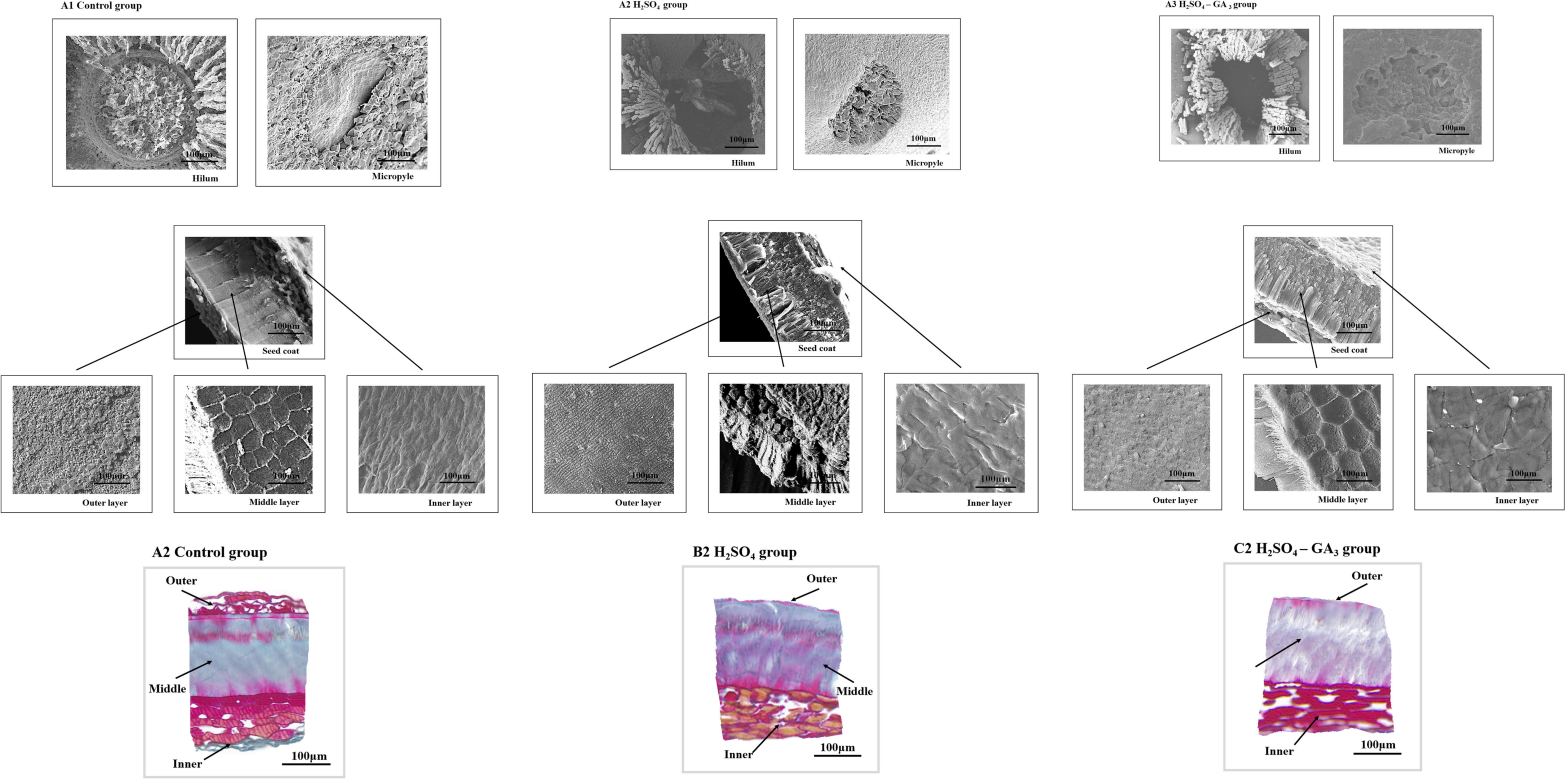
Three-dimensional cellular structure of the outer surface of the outer seed coat in the H2SO4 treatment group (0, 50, and 70 days) **(A)** and in the H2SO4-GA3 treatment group (0, 30, and 45 days) **(B)** during the cold stratification period.

**Figure 8:**
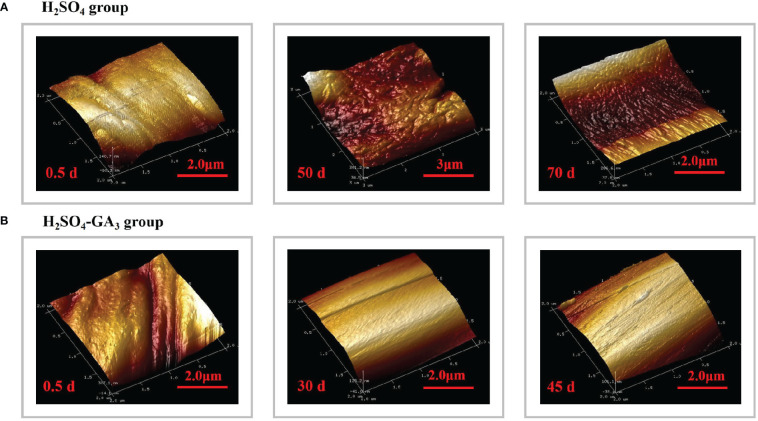
Three-dimensional cellular structure of the outer surface of the outer seed coat in the H_2_SO_4_ treatment group (0, 50, and 70 days) **(A)** and in the H_2_SO_4_-GA_3_ treatment group (0, 30, and 45 days) **(B)** during the cold stratification period.

In **Figure 1**, percentage germination and water content data were analyzed by SPSS statistics 27.0. One-way ANOVA was used to determine significant differences between each treatment (p< 0.05). Pearson’s correlation coefficient was used to determine correlation between each treatment. **Figure 1** was drawn by Origin 2021 software (the software for drawing and data analysis developed by OriginLab). In **Figures 2**–**4**, water content, signal-to-noise ratio (SNR), hardness, content of lignin, cellulose, hemicellulose, and total phenols data were analyzed by MATLAB2017b software (the software for analyze data, develop algorithms, and create mathematical models). One-way ANOVA was used to determine significant differences between each treatment (p< 0.05). Least squares was used to determine the fitted model. **Figures 2**–**4** were drawn by MATLAB2017b software. In **Figures 5**–**7**, the Image J software was used to acquire the MR image slices, SEM images, and PSD images. In **Figure 8**, the NanoScope Analysis v1.40r1 software (the software for store, share, view, and analyze 3D images from profilometers, AFMs, and other 3D microscopes) was used to acquire the 3D AFM images.

## 3 Results

### 3.1 Germination of control, H_2_SO_4_, and H_2_SO_4_-GA_3_-treated seeds during cold stratification period

During cold stratification process, at 15, 30, and 45 d, no seed germinated in neither control nor H_2_SO_4_-treated groups. In the control group, there was still no seed germination at 60 d, and only a small number of seeds germinated at 75 d, with a germination percentage of 0.31 ± 0.0009%. A small amount of H_2_SO_4_-treated seeds germinated (0.31 ± 0.0073%) at 60 d, and the germination percentage was 19.71 ± 0.0242% at 75 d. It can be seen that the dormancy of the control and H_2_SO_4_-treated seeds was not breaking. Seeds of H_2_SO_4_-GA_3_-treated groups germinated (14 ± 0.02%) at 15 d. Germination percentage reached 43% ± 0.02% at 30 d. At 45 d, germination percentage had exceeded 50%, which was 53% ± 0.01%. At 60 d, germination percentage had exceeded 60%, which was 61% ± 0.01%. At 75 d, germination rate was close to 70%, which was 67% ± 0.01% (**Figure 1**). Therefore, dormancy cannot be broken if only treated with H_2_SO_4_. Seed germination occurs only when H_2_SO_4_ and GA_3_ are combined.

### 3.2 Dynamic changes in the seed water content during the imbibition and cold stratification periods

#### 3.2.1 Seed water content

Water is vital for seed germination (Bradford, 1990). It is also critical for seed physiological and biochemical activities. After sufficient water has been absorbed, a series of essential processes occurs within the seed, ultimately leading to germination. The seed water content for the control, H_2_SO_4_, and H_2_SO_4_-GA_3_ treatment groups differed significantly in the imbibition (**Figure 2A**) and cold stratification (**Figure 2B**) periods. During the 180-h imbibition period, the water content increased slowly in the control group, reaching 21.81% ± 1.79%. In contrast, at the 50-h time point, the water contents in the H_2_SO_4_ and H_2_SO_4_-GA_3_ groups were 40.54% ± 0.92% and 47.53% ± 0.40%, respectively. At 180 h, the water contents in the H_2_SO_4_ and H_2_SO_4_-GA_3_ groups were 45.28% ± 1.82% and 49.25% ± 0.23%, respectively (no significant difference), which were significantly higher than the water content of the control group.

During the cold stratification period, the water content of the control group remained low (19.35% ± 0.09% at 75 days), while the water contents of the H_2_SO_4_ and H_2_SO_4_-GA_3_ groups increased rapidly during the early stage, reaching 48.87% ± 0.66% and 42.83% ± 0.11%, respectively, at 15 days. The water contents of the H_2_SO_4_ and H_2_SO_4_-GA_3_ groups at the later time points were, respectively, 54.77% ± 0.10% and 52.01% ± 0.67% (at 45 days) and 74.09% ± 0.66% and 69.68% ± 0.68% (at 75 days). The trends in the water contents of the three treatment groups were similar between the cold stratification and imbibition periods. In addition, the water content was significantly higher for the H_2_SO_4_ and H_2_SO_4_-GA_3_ groups than for the control group, indicative of the water permeability barrier in the seed coat. After the H_2_SO_4_ or H_2_SO_4_-GA_3_ treatment, the water permeability barrier of the seed coat was eliminated and free water quickly entered the seeds. Although the water contents of the H_2_SO_4_ and H_2_SO_4_-GA_3_ groups were sufficient for germination at 15 days, only the seeds in the H_2_SO_4_-GA_3_ group germinated. Thus, even if the increase in the water content due to the elimination of the seed coat permeability barrier by the H_2_SO_4_ treatment was sufficient for germination, the seeds germinated only when GA_3_ was applied as well.

#### 3.2.2 Distribution of water in seeds

The MRI images revealed the distribution of water in the seeds in the imbibition and cold stratification periods (**Figure 5**). During the imbibition period, water (red signal) was detected in the radicle much later in the control and H_2_SO_4_ groups (40 h) than in the peeled and H_2_SO_4_-GA_3_ groups (0 h). As the imbibition continued, the number of red water signals increased for the seeds in each treatment group. However, there were significant differences in how quickly the red water signals appeared and in the size of the area with red water signals among the treatment groups. When the control seeds were imbibed for 160 h, the red water signal was undetectable in the seed coat, suggestive of a water permeability barrier. Compared with the control seeds, the seeds in the peeled, H_2_SO_4_, and H_2_SO_4_-GA_3_ groups absorbed water more quickly. Interestingly, the water uptake rate was significantly higher in the peeled and H_2_SO_4_-GA_3_ groups than in the H_2_SO_4_ group. After imbibing for 160 h, red water signals were detected in most areas of the radicle, cotyledon, outer endosperm, and inner endosperm, but not in the seed coat, in the peeled and H_2_SO_4_-GA_3_ groups. In contrast, red water signals were undetectable in the inner endosperm and seed coat in the H_2_SO_4_ group. These observations suggest that there is a water permeability barrier in the seed coat and GA_3_ significantly enhances the ability of seeds to absorb water.

The H_2_SO_4_ and H_2_SO_4_-GA_3_ treatments accelerated the release of seed dormancy during the cold stratification period. However, the rate of dormancy release differed between the two treatment groups, as did the timing of the appearance and the intensity of the red water signals within the seeds. At the beginning of the cold stratification period, red water signals were detected in the radicles of the control, H_2_SO_4_, and H_2_SO_4_-GA_3_ groups. On day 30, the red water signal was observed only in the inner endosperm of the H_2_SO_4_-GA_3_ group. On day 45 (i.e., late dormancy release stage in the H_2_SO_4_-GA_3_ group), red water signals were detected in more areas within the seed. More specifically, they were mainly distributed in the radicle, but they were also detected in the cotyledon and inner and outer endosperm. There were no significant changes in the red water signals in the control and H_2_SO_4_ groups. On day 70 (i.e., early dormancy release stage in the H_2_SO_4_ group), there was a marked increase in the number of areas containing red water signals within the seeds. This period coincided with the germination stage of the H_2_SO_4_-GA_3_ group, in which extremely prominent red water signals were detected throughout the seed interior.

#### 3.2.3 Signal-to-noise ratio data for different seed tissues

During the imbibition (**Figure 3I**) and cold stratification (**Figure 3II**) periods, the SNR data for the hilum were consistently lower for the H_2_SO_4_-GA_3_-treated seeds than for the control and H_2_SO_4_-treated seeds. In contrast, the SNR data for the micropyle, endosperm near the micropyle, radicle, cotyledon, and endosperm near the hilum were higher in the H_2_SO_4_-GA_3_ group than in the other two groups. Accordingly, the hilum may not be associated with a water gap. Notably, the SNR data for the hypocotyl during part of the imbibition period (60–180 h) and part of the cold stratification period (15–75 days) and for the micropyle during part of the cold stratification period (45–75 days) were significantly higher for the H_2_SO_4_-GA_3_-treated seeds than for the control and H_2_SO_4_-treated seeds. Hence, the permeability at the radicle, cotyledon, and endosperm near the micropyle of the H_2_SO_4_-GA_3_ seeds increased significantly, especially at the hypocotyl and micropyle. The hypocotyl is positioned close to the micropyle. Accordingly, water may gradually spread (radicle→hypocotyl→cotyledon→endosperm) through the micropyle.

### 3.3 Two- and three-dimensional characterization of the cells at the seed coat surface during the cold stratification period

In the SEM images, the hilum and micropyle of the control seeds were clearly visible. The circular hilum was detected at the top of the seeds. The cells on the inner side of the circular structure were uneven and densely arranged. The oval micropyle, which was detected at the bottom of the seeds, consisted of irregular cells that were tightly arranged. The SEM and PSD images showed that the seed coat was composed of outer, middle, and inner layers. The outer layer was a cuticle with densely arranged cells. The middle layer consisted of columnar cells, which were densely arranged in parallel. The inner layer comprised irregular tetragonal cells with an uneven surface. After the H_2_SO_4_ treatment, the hilum consisted of acid-etched and pore-like cells. Pores that varied in size were detected at the micropyle. The long strip-shaped quadrilateral cells on the outer surface of the outer layer were arranged to form a gully that was covered by a few small holes. A few small holes were also detected at the surface of the inner seed coat. Following the H_2_SO_4_-GA_3_ treatment, the changes in the hilum and micropyle were almost identical to those observed in the H_2_SO_4_ group, suggestive of the opening of water gaps at these two points. The outer surface of the outer layer was densely covered with small pores. There were obvious gaps between the irregular quadrilateral cells on the outer surface of the inner layer. No significant changes were detected in the middle layer of the H_2_SO_4_ and H_2_SO_4_-GA_3_ samples (**Figure 6**).

During the cold stratification period, there were significant changes in the seed coat cellular structures in the H_2_SO_4_ and H_2_SO_4_-GA_3_ treatment groups (**Figure 7**). The main features were the pores in the outer seed coat and the gaps between the inner seed coat cells. The number and size of the pores gradually increased as seed dormancy was released. The changes in the pores on the surface of the inner seed coat occurred earlier in the H_2_SO_4_-GA_3_ group than in the H_2_SO_4_ group, and there were fewer pores in the H_2_SO_4_-GA_3_ group than in the H_2_SO_4_ group. On day 30, the cells of the outer layer in the H_2_SO_4_ and H_2_SO_4_-GA_3_ groups were bulging, and some of them had ruptured. Gaps appeared between the cells of the inner layer in the H_2_SO_4_-GA_3_ group, but only small holes were detected in the H_2_SO_4_ group. By day 45 of the cold stratification period, dormancy was broken for most of the seeds in the H_2_SO_4_-GA_3_ group. Grooves appeared between the cells of the outer seed coat. The inner seed coat was densely packed with pores and had a smooth surface. Similar changes were observed in the seed coat of the H_2_SO_4_ group on day 70. During the cold stratification period, the inner and outer seed coats of the control group did not change significantly. Moreover, holes and cracks were undetectable. Thus, the H_2_SO_4_ treatment appeared to disrupt the seed coat structure, thereby enhancing the permeability of the seed coat. The seed coat changes occurred earlier and were more pronounced after the H_2_SO_4_-GA_3_ treatment than after the H_2_SO_4_ treatment. Therefore, the cellular changes in the seed coat are likely closely related to dormancy release.

The AFM results revealed the significant differences in the three-dimensional morphology of the cells on the outer surface of the outer seed coat between the H_2_SO_4_ and H_2_SO_4_-GA_3_ treatment groups (**Figure 8**). At the beginning of the cold stratification period (day 0), the outer surface cells of the H_2_SO_4_-GA_3_ group were relatively smooth, with dimples and bumps that varied in size. In the H_2_SO_4_ group, the outer surface of the outer seed coat was densely packed with small and long cells that were folded and convex. On days 30 and 50 of the cold stratification period, the cells on the surface of the H_2_SO_4_-GA_3_-treated samples were obliquely raised and the cell surface was smooth, whereas the cells on the outer surface of the outer seed coat of the H_2_SO_4_ group were clustered in blocks of varying sizes, with a high and undulating appearance. On days 45 and 70 of the cold stratification period, the cells on the outer surface of the outer seed coat of the H_2_SO_4_-GA_3_ group had large oblique bumps with densely packed dimples of various sizes. The cells on the outer surface of the outer seed coat of the H_2_SO_4_ group were raised slightly in long strips of varying sizes.

### 3.4 Changes in the mechanical properties of the seed coat during the cold stratification period

The seed coat hardness increased and decreased significantly in the H_2_SO_4_ and H_2_SO_4_-GA_3_ treatment groups, respectively (**Figure 8**). The initial seed coat hardness (day 0) of the H_2_SO_4_ group was 9.48 HV, but it increased to 9.63 HV on day 50 and to 12.73 HV on day 70. The seed coat hardness of the H_2_SO_4_-GA_3_ group was initially 10.38 HV (day 0) and then decreased to 8.40 HV on day 50 and to 7.80 HV on day 70. These findings suggest that the exogenous application of GA_3_ may have contributed to the decrease in the seed coat hardness.

During the cold stratification period, in the H_2_SO_4_ and H_2_SO_4_-GA_3_ treatment groups, the cellulose content increased and then decreased, while the total phenol content tended to decrease. However, the two treatment groups differed regarding the changes in the lignin and hemicellulose contents. The lignin content decreased and then increased in the H_2_SO_4_ group, whereas it decreased continuously in the H_2_SO_4_-GA_3_ group. The hemicellulose content tended to increase in the H_2_SO_4_ group, while it decreased and then increased in the H_2_SO_4_-GA_3_ group (**Figure 4**). Accordingly, GA_3_ significantly decreased the lignin and hemicellulose contents, possibly *via* accelerated degradation.

### 3.5 Changes in the composition of the seed coat during the cold stratification period

Among the compounds detected by the LC-MS analysis of the seed coat in the H_2_SO_4_ treatment group (0, 50, and 70 days), 789 matched the compounds in the mzCloud database (**Table 2**). The following six compounds were identified among the top 10 peaks at all three time points: erucamide, linoleic acid, 1-linoleoyl glycerol, 2-arachidonoyl glycerol, oleic acid, and (+/−)12(13)-DiHOME. The other four identified compounds on day 0 were bis(2-ethylhexyl) phthalate, dibutyl phthalate, 9-oxo-10(E),12(E)-octadecadienoic acid, and stearamide. The other four identified compounds on day 50 were 2-(14,15-epoxyeicosatrienoyl) glycerol, α-eleostearic acid, (+/−)12-HpETE, and polyethylene glycol (PEG) n10. The other four identified compounds on day 70 were bis(2-ethylhexyl) phthalate, α-eleostearic acid, prostaglandin H, and betaine.

**Table 2:** The contents of the compounds identified in the seed coat of the H_2_SO_4_ and H_2_SO_4_-GA_3_ treated groups during stratification process (display-split by area).

A H_2_SO_4_ group - Chemical substance name			
Number	Time		
	0d	50d	70d
1	Erucamide	Erucamide	Erucamide
2	Linoleic acid	Linoleic acid	Linoleic acid
3	1-Linoleoyl glycerol	1-Linoleoyl glycerol	1-Linoleoyl glycerol
4	2-Arachidonoyl glycerol	2-Arachidonoyl glycerol	2-Arachidonoyl glycerol
5	Oleic acid	Oleic acid	Oleic acid
6	(+/-)12(13)-DiHOME	(+/-)12(13)-DiHOME	(+/-)12(13)-DiHOME
7	Bis(2-ethylhexyl) phthalate	2-(14,15-Epoxyeicosatrienoyl) glycerol	Bis(2-ethylhexyl) phthalate
8	Dibutyl phthalate	α-Eleostearic acid	α-Eleostearic acid
9	9-Oxo-10(E),12(E)-octadecadienoic acid	(+/-)12-HpETE	Prostaglandin H1
10	Stearamide	PEG n10	Betaine
B H_2_SO_4_-GA_3_ group - Chemical substance name			
Number	Time		
	0d	30d	45d
1	Erucamide	Erucamide	Erucamide
2	Gibberellic acid	Gibberellic acid	Gibberellic acid
3	Indole-3-acrylic acid	α-Lactose	α-Lactose
4	Oleamide	2,2'-Methylenebis(4-methyl-6-tert-butylphenol)	2,2'-Methylenebis(4-methyl-6-tert-butylphenol)
5	(+/-)12(13)-DiHOME	Dibutyl phthalate	Dibutyl phthalate
6	Stearic acid	PEG n10	PEG n10
7	Maltotetraose	PEG n11	PEG n11
8	Hexadecanamide	PEG n12	PEG n12
9	Bis(2-ethylhexyl) phthalate	Nicotinamide	Myristyl sulfate
10	Dibutyl sebacate	Stearamide	α-Pyrrolidinopentiothiophenone

A total of 483 compounds detected in the seed coat of the H_2_SO_4_-GA_3_ treatment group (0, 30, and 45 days) matched compounds in mzCloud. The two compounds identified among the top 10 peaks at all three time points were erucamide and gibberellic acid. The other eight identified compounds on day 0 were indole-3-acrylic acid, oleamide, (+/−)12(13)-DiHOME, stearic acid, maltotetraose, hexadecanamide, bis(2-ethylhexyl) phthalate, and dibutyl sebacate. The other eight identified compounds on day 30 were α-lactose, 2,2′-methylenebis(4-methyl-6-tert-butylphenol), dibutyl phthalate, PEG n10, PEG n11, PEG n12, nicotinamide, and stearamide. The other eight identified compounds on day 45 were the same as those identified on day 30, with the exception of myristyl sulfate and α-pyrrolidinopentiothiophenone.

## 4 Discussion

### 4.1 Weakening of the permeability barrier in response to the H_2_SO_4_-GA_3_ treatment

Water uptake is the crucial first step of the seed germination process. The water content increased significantly in the control, H_2_SO_4_, and H_2_SO_4_-GA_3_ treatment groups during the early stage (0–15 days) of the cold stratification period. There was also a significant increase in the water content of the H_2_SO_4_ and H_2_SO_4_-GA_3_ groups in the later stage of the cold stratification period (45–75 days), which provided the water needed for embryo growth. The correlation analysis indicated that the water content and the seed germination rate were significantly positively correlated in the H_2_SO_4_-GA_3_ group (**Table 3**). The initial seed moisture content in the H_2_SO_4_-GA_3_ group was 34%. A few seeds started to germinate when the moisture content reached 35%–45%. The germination rate increased significantly when the moisture content reached 45%–55%. More than 50% of the seeds germinated when the water content reached 55%–75%. Gibson and Bachelard (1986) determined that *Eucalyptus sieberi* L. Johnson seeds can germinate normally within 60 h at 15–20°C when the seed moisture content exceeds 30%. In an earlier study, Zhang (2018) confirmed the positive correlation between the germination rate and the moisture content of sunflower seeds. The sunflower seed germination rate was 96% within 50 days after the moisture content reached 5.8%–6.8%. When the moisture content decreased below 5.5%, it needed to be increased to 5.8%–6.8% to ensure the seeds germinated normally. We speculate that during the imbibition and stratification periods, the hydration of *T. miqueliana* seeds gradually increases to the threshold level required for germination, while basic metabolic processes are activated, ultimately leading to seed dormancy release. Interestingly, similar trends in the changes to the water content were observed for the H_2_SO_4_ and H_2_SO_4_-GA_3_ treatment groups. However, because of a lack of accelerated endosperm hydration and endosperm and seed coat metabolism due to the absence of GA_3_, the germination rate of the H_2_SO_4_ group remained low.

**Table 3:** Correlation between the water content and germination rate in the H_2_SO_4_ and H_2_SO_4_-GA_3_ treatment groups during the cold stratification period.

		Germination percentage		
		Control group	H_2_SO_4_ group	H_2_SO_4_-GA_3_ group
Water content	Control group	0.443 (p=0.379)	–	–
	H_2_SO_4_ group	–	0.770 (p=0.073)	–
	H_2_SO_4_-GA_3_ group	–	–	0.909 (p=0.012)

Some impermeable seeds have specific sites on their seed coat that control water entry (e.g., hilum, lens, and/or micropyle) (Kelly et al., 1992). The parts controlling water entry vary among seeds. For example, the micropyle is the water control site of the seeds produced by certain leguminous and *Anacardiaceae* species (Pell et al., 2011; Luan et al., 2017). The hilum is the water control site of cotton seeds (Egley, 1989), whereas the chalaza is the water control region of *Sida spinosa* seeds (*Malvaceae*) (Egley et al., 1986). There are two types of inlet holes in impermeable hard-coat seeds. Specifically, one is situated close to the micropyle (a small opening and remnant of the embryo sac) and is sometimes naturally closed by a waxy “lid” (Saio, 1976). The other is at the strophiole (i.e., raphe or lens), which is a predetermined and inherently weak part of the seed coat located on the other side of the hilum (Hopkinson, 1993).

The hilum of *T. miqueliana* seeds is rounded, and the outer surface is densely covered with lamellate cells. The micropyle is oval and has a raised caruncle on the outer surface. The SEM results showed that the H_2_SO_4_ and H_2_SO_4_-GA_3_ treatments disrupted the seed coat structure by unblocking (i.e., opening) the micropyle and hilum. The NMR images and SNR data indicated that water entered the seed cavity through the micropyle rather than the hilum or seed coat. There may be water channels in the endosperm close to the micropyle that allow water to enter the seed cavity. The red water signal appeared fastest and covered the largest region in the H_2_SO_4_-GA_3_ treatment group, followed by the H_2_SO_4_ treatment group and then the control group. The H_2_SO_4_ treatment promoted the entry of water at the micropyle of *T. miqueliana* seeds, resulting in a rapid increase in the water level in the seed interior. The addition of GA_3_ accelerated the hydration of the endosperm to the threshold required for germination.

Notably, although the cellular structure in random areas of the seed coat was obviously damaged, the entry of water was undetectable in those areas. Similar findings were reported for cotton seeds. Simpson et al. (1940) analyzed five seed lots (acid-delinted and untreated seeds) and detected similarities in water absorption. When both ends of the seeds were sealed, the outer colorless and palisade layers remained unstained, implying that water did not readily pass through the side walls. Because of its high mechanical strength, the hard *T. miqueliana* seed coat does not crack under normal conditions. Even when the seed coat cell structure is destroyed, water still enters *via* the micropyle and not through the seed coat.

### 4.2 Effect of the H_2_SO_4_-GA_3_ treatment on the cell structural and mechanical properties of the seed coat

Changes in seed coat hardness are important for seed germination. Garcıía-Gusano et al. (2004) determined that endocarp hardness has a significant effect on almond seed germination. Eliminating the endocarp can shorten the stratification period necessary for the germination of hard-shelled cultivars by 3 weeks. During the cold stratification period, the *T*. *miqueliana* seed coat hardness increased significantly in response to the H_2_SO_4_ treatment, which was in contrast to the observed decrease following the H_2_SO_4_-GA_3_ treatment. In this study, the dormancy of the *T*. *miqueliana* seeds was not released in the H_2_SO_4_ group, whereas the dormancy of the seeds was rapidly broken by the H_2_SO_4_-GA_3_ treatment. The correlation analysis revealed a significant negative correlation between the germination rate and the seed coat hardness in the H_2_SO_4_-GA_3_ group (**Supplementary Figure S1**). Accordingly, seed coat hardness is an important factor influencing the germination of *T. miqueliana* seeds. A significant decrease in the seed coat hardness contributes to MD release.

The H_2_SO_4_-GA_3_ treatment altered the contents of compounds related to seed coat mechanical properties. More specifically, there was a positive correlation between the seed coat hardness and the lignin and total phenol contents (**Supplementary Figure S1**). Lignin is a phenolic compound that increases the compressive strength and stiffness of the cell wall. Zhang et al. (2015) reported that seed hardness is positively correlated with the seed coat lignin content of six pomegranate varieties. Marbach and Mayer (1974) demonstrated that the permeability of the *Pisum elatius* seed coat to water is related to the abundance and oxidation of phenolic compounds in the seed coat; impermeable seed coats have a high phenolic content. The H_2_SO_4_-GA_3_ treatment may have accelerated the degradation of lignin and phenolics, which subsequently decreased the mechanical constraint and adversely affected the permeability barrier of the seed coat.

### 4.3 Effect of the H_2_SO_4_-GA_3_ treatment on the changes to the seed coat components after permeability increased

The changes in the *T. miqueliana* seed coat components following the increase in permeability indicated that although both treatments (H_2_SO_4_ and H_2_SO_4_-GA_3_) opened the water gap (micropyle), there were significant differences in the seed coat composition between the two treatment groups during the cold stratification period. With the exception of erucamide, (+/−)12(13)-DiHOME, bis(2-ethylhexyl) phthalate, and PEG n10, the identified compounds among the top 10 peaks for the seed coats in the H_2_SO_4_ and H_2_SO_4_-GA_3_ treatment groups differed during the cold stratification period. Notably, indole-3-acrylic acid (member of the IAA family) was detected in the seed coat of the H_2_SO_4_-GA_3_ group on day 0. Earlier research indicated IAA controls plant developmental processes (Kaldewey, 1969). Indole-3-acetic acid (4 ppm and 5 ppm) can rapidly break the dormancy of *Lablab purpureus* L. seeds, with germination rates reaching 70% (https://edupediapublications.org/journals). We speculate that the IAA that was detectable in the seed coat after the H_2_SO_4_-GA_3_ treatment may contribute to seed dormancy release.

Interestingly, PEG n10, PEG n11, and PEG n12 were among the compounds identified in the seed coat of the H_2_SO_4_-GA_3_ group on days 30 and 45. Because PEG is a flexible and water-soluble polymer, it can be used to create very high osmotic pressures (https://en.wikipedia.org/wiki/Polyethylene_glycol), which induce the rapid flow of water inside and outside the cell membrane. The absorption of water necessary for seed germination depends on an imbalance in the water potential between the seed and its external environment (Yang et al., 2010). Bradford (1986) reported that the germination of lettuce seeds under stress conditions may be improved by osmotically regulating the water relations inside and outside of the seed. When the water absorption due to an osmotic gradient leads to radicle expansion, seeds will begin to germinate. Many types of PEGs were identified in the H_2_SO_4_-GA_3_ group, but not in the H_2_SO_4_ group. These PEGs may disrupt the water potential balance between the internal and external environments of the seed. This imbalance may result in the absorption of a large amount of water, which will quickly hydrate the endosperm and break the seed dormancy.

## 5 Conclusion

In this study, the H_2_SO_4_-GA_3_ treatment rapidly released the PY + MD + PD of *T. miqueliana* seeds. First, it opened the water gap (micropyle) and allowed water to enter the seed cavity along a specific channel (micropyle→radicle→hypocotyl→cotyledon→endosperm), thereby eliminating the permeability barrier of the seed coat. Second, it was crucial for weakening the seed coat because it accelerated the degradation of lignin and phenolics and decreased the mechanical constraint of the hard seed coat. Third, it promoted the metabolism of seed coat components. Different types of PEG increased the speed at which water entered the seed cavity and accelerated hydration and the effects of GA_3_ on the endosperm, thereby rapidly breaking the PD associated with the endosperm.

## Data availability statement

The datasets presented in this study can be found in online repositories. The names of the repository/repositories and accession number(s) can be found in the article/**Supplementary Material**.

## Author contributions

YBS, and YW conceived and designed the experiments; YW, CYP, and MZW performed the experiments; YW, XRS, and QG analyzed the data; WHH, YBS, AMW, HWP, and ZYD reviewed and edited the paper.
