# Supplementary material for: Effects of H2SO4, GA3, and cold stratification on the water content, coat composition, and dormancy release of Tilia miqueliana seeds
Source: Front Plant Sci. 2023 Nov 9;14:1240028. doi: 10.3389/fpls.2023.1240028 (PMC10699446; doi:10.3389/fpls.2023.1240028)
Supplement: Supplementary file 1 [file Image_1.pdf]

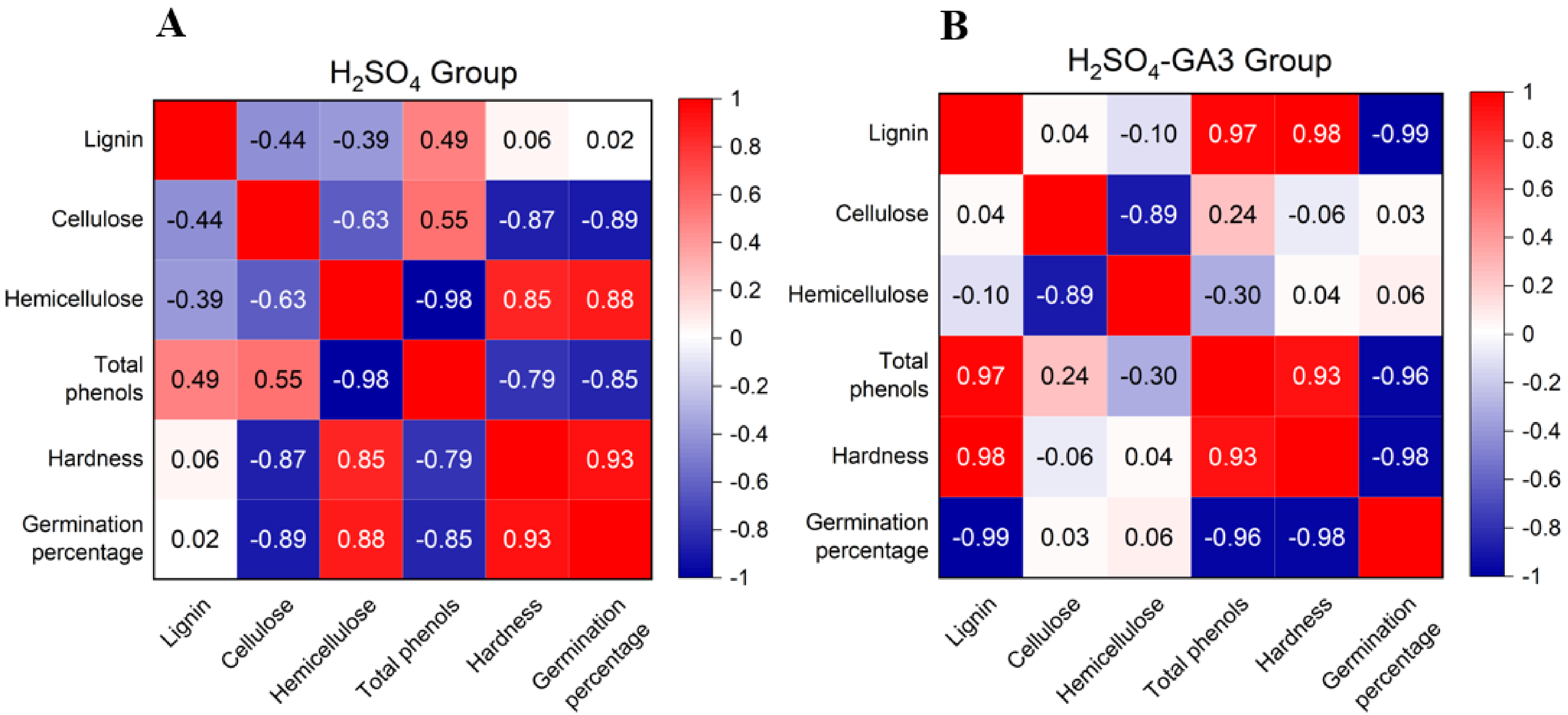

Fig. 1. Correlation analysis of lignin, cellulose, hemicellulose, total phenol content, seed coat hardness with germination percentage in H_2SO_4 and H_2SO_4-GA_3 treated groups during cold stratification.
